# Population‐Based Cosmetics Exposure Parameters: Survey Approaches and Cosmetics Usage Results From Different Countries

**DOI:** 10.1111/jocd.70471

**Published:** 2025-09-30

**Authors:** Xinxin Yin, Ziyin Li, Jiajia Fu, Weizuo Liao, Jiangying Yan, Weiliang Wu, Zhini He, Feifei Xu, Jun Yan, Jia Song, Xianwu Peng, Xingfen Yang

**Affiliations:** ^1^ Food Safety and Health Research Center, NMPA Key Laboratory for Safety Evaluation of Cosmetics, Guangdong Provincial Key Laboratory of Tropical Disease Research, School of Public Health Southern Medical University Guangzhou People's Republic of China; ^2^ Amway (China) Co, Limited Guangzhou People's Republic of China; ^3^ China Association of Fragrance Flavour and Cosmetic Industries Beijing People's Republic of China

**Keywords:** cosmetics consumption, investigation approaches, risk assessment, survey approaches, usage patterns

## Abstract

**Background:**

Access to reliable exposure data is critical for conducting risk assessments of ingredients in cosmetic products. Cosmetic usage patterns, including product types, user percentages, daily product numbers, amounts, and frequency of use, can vary significantly across regions, countries, and populations. Therefore, obtaining comprehensive cosmetic usage data and establishing a robust database of exposure parameters are essential for accurately assessing the exposure levels of cosmetic ingredients.

**Problem Statement:**

Despite the critical need for exposure data, a systematic review analyzing existing survey approaches and methodologies for cosmetic usage has been lacking.

**Objectives:**

This study aimed to compare cosmetic usage patterns across different regions and countries, systematically summarize survey approaches and methodologies, and obtain accurate and effective cosmetic exposure assessment parameters to facilitate the risk assessment of cosmetic products.

**Methods:**

A systematic review approach was used following the Preferred Reporting Items for Systematic Reviews and Meta‐analyses (PRISMA) guidelines. PubMed and CNKI databases were retrieved in April 2025 for articles published between 2005 and 2025. With a total of 38 publications deemed relevant based on the established inclusion criteria, the following information was extracted: type of study, characteristics of the population (number, age, sex, and region of origin), period of data collection, types of products studied, methods of data collection, and consumption and/or exposure parameters obtained.

**Results:**

This study reviews surveys on cosmetics consumer exposure parameters conducted by the European Union, the United States, and some Asian countries over the past few decades. It analyzes the differences in research on cosmetics usage patterns across these regions/countries and summarizes domestic and international investigation approaches for cosmetic consumption surveys, including their advantages, disadvantages, and appropriate applications.

The results demonstrated the current status of cosmetic usage pattern surveys in different countries/regions. There are differences in cosmetic usage patterns among countries in the European Union, the United States, and some Asian countries. Currently, cosmetics consumption data are mainly divided into qualitative data and quantitative data. Qualitative data include types of products, user usage, usage location, etc. Quantitative data comprise the percentage of users, frequency of use, and amount of cosmetics used (per use and per day), etc. These data were obtained through methods such as questionnaires, weighing approaches, etc. During the investigation, appropriate methods were selected to obtain the corresponding types of data.

**Significance:**

The findings of this study will provide a valuable reference for initiating future investigations of cosmetics usage and provide a model for subsequent assessments of population exposure to ingredients in cosmetic products.

**Abbreviations**
ANSM: The French National Agency for Drugs and Health Product Safety
COLIPA: The European Cosmetics, Toiletry and Perfume Association
ETCD: European Toiletries and Cosmetics Database
EU: The European Commission Regulation
FDA: The US Food and Drug Administration
ISC: The Ian Smith Consultancy
JCIA: The Japan Cosmetics Industry Association
LERCCo: The Laboratoire d'Evaluation du Risque Chimique pour le Consommateur
MoCRA: The modernization of cosmetics Regulation Act
MoS: Margin of safety
NOAEL: No Observable Adverse Effect Level
PAL: Pharmaceutical Affairs Law
PCPC: The Personal Care Products Council
REACH: Registration, Evaluation, Authorisation and Restriction of Chemicals
RIVM: The National Institute for Public Health and the Environment
SCCS: The Scientific Committee on Consumer Safety
SED: Systemic exposure dose
SIFBI: The Singapore Food and Biotechnology Innovation Research Institute

## 1 Introduction

The European Commission Regulation (EU) 2023/1490 mandates that cosmetic products must be safe for human health when used under normal or reasonably foreseeable conditions. To perform a safety evaluation, comprehensive usage data for finished cosmetic products are necessary to assess consumer exposure to cosmetic ingredients. According to the US Food and Drug Administration (FDA), a cosmetic is defined as “a product (excluding pure soap) intended to be applied to the human body for cleansing, beautifying, promoting attractiveness, or altering the appearance” [1]. In China, the Regulations on Supervision and Administration of Cosmetics (Decree No. 727 of the State Council of the People's Republic of China) were promulgated on June 29, 2020. For the purpose of this Regulation, “cosmetics” are defined as daily‐use chemical products applied to the surface of any part of the human body, such as skin, hair, nails, and lips, by way of smearing, spraying, or other similar methods for the purposes of cleaning, protection, beautification, and modification [2]. These regulations are designed to regulate the production and distribution of cosmetics, strengthen their supervision and administration, ensure product quality and safety, safeguard consumer health, and foster the healthy development of the cosmetics industry.

Cosmetic products are widely used across most regions and cultures worldwide, primarily driven by consumer interest in personal appearance, hygiene, and well‐being. As consumer goods that address the public's needs for an improved quality of life, cosmetics concretely reflect evolving expectations for health and beauty. In 2020, the global cosmetics market experienced a contraction due to the COVID‐19 pandemic, with market growth declining by 3.92%. However, the overall market size remained above $480 billion, exceeding the market size observed in 2017. From 2021 to 2022, the global cosmetics market size rapidly rebounded, reaching $565.2 billion in 2022, the highest value in nearly a decade. As the world's second‐largest consumer market for cosmetics, the retail sales of cosmetics in China reached 455.3 billion yuan, with consumer goods accounting for over 1% of the national retail sales, and the annual per capita consumption of cosmetics exceeding 300 yuan in 2021. The compound annual growth rate from 2023 to 2027 is projected to reach 5.91%, with the market size expected to reach 728.8 billion yuan by 2027 [3].

The rapid expansion of the global cosmetics market requires high‐quality development, industrial transformation, and upgrading within the sector. This process requires further in‐depth research into the fundamental technical system for cosmetics standards, safety assessment, and risk monitoring. During the process of constructing a fundamental technical system for cosmetic safety assessment, it is essential to comprehensively and systematically build a cosmetics exposure parameters database for consumers, which will fill existing gaps in cosmetics usage data, promote the risk management level in the cosmetics industry across various regions and countries, and enhance the modern governance capabilities pertaining to cosmetics.

Risk assessment is an effective means of product safety assessment and is crucial for the long‐term development of the cosmetic industry. Accordingly, conducting risk assessment on cosmetic products is essential to ensure consumer health. The traditional risk assessment paradigm is based on four steps: “hazard identification, dose‐response assessment, exposure assessment, and risk characterization.”

The safety assessment of cosmetic products should be exposure‐oriented, integrating consumer use practices to ensure product safety. For the safety assessment of cosmetic ingredients, the Scientific Committee on Consumer Safety (SCCS) considers all available scientific data while adhering to the testing and marketing bans stipulated by Regulation (EC) No. 1223/2009. This includes evaluating the physical and chemical properties of the compounds under investigation, exposure via relevant exposure routes, and *in silico* data. Exposure data for the finished product are essential for determining the systemic exposure dose (SED) for each ingredient. This SED value is subsequently used to calculate the margin of safety (MoS), which represents the ratio between a No Observable Adverse Effect Level (NOAEL) and the SED value. Exposure parameters, as a means of quantitatively and qualitatively evaluating harmful factors in cosmetics, are key parameters in human exposure and health risk assessment. Their accuracy is a critical determinant of the accuracy and scientific validity of health risk assessments. Therefore, investigating cosmetics consumption is an indispensable and key step that requires an understanding of consumers' habits and practices in using various product categories. At the same time, consumption data for finished cosmetic products (including frequency of use, amount per use, amount per day) are necessary to assess the corresponding consumer exposure (mg/kg bw/day) [4]. Currently, published or otherwise readily available exposure data for cosmetic products are limited. When conducting risk assessments, the use of appropriate exposure assessment approaches can systematically yield basic data on the cosmetic product type(s), application method, ingredient concentration, quantity used per application, frequency of use, and duration of exposure of ingredients in cosmetics to the human body, in order to determine the exposure dose of ingredients in cosmetic products and evaluate the health effects of cosmetics through all relevant routes of exposure, such as dermal, oral, and inhalation. Therefore, a clear understanding of cosmetic use behavior within a specific country/area derived from population‐based usage pattern studies is essential.

During the first decade of the 21st century (2000–2010), the European Union and the United States conducted large‐scale studies on cosmetics usage in their respective regions. Since 2010, countries such as France, the Netherlands, Japan, China, and Korea have also conducted surveys on the use of cosmetics among their respective populations. Most of these surveys have been limited to specific regional populations and often lack comprehensive full‐category coverage of cosmetic products, preventing real‐world population exposures. Therefore, a dedicated project focusing on a nationally representative survey of cosmetic usage is essential. Such an initiative aims to ascertain the realistic exposure levels of cosmetics among local consumers. The evaluation results obtained can scientifically guide the scientific supervision of cosmetics and address the needs of modern local cosmetics regulation.

## 2 Methods

### 2.1 Databases and research equations

**FIGURE 1:**
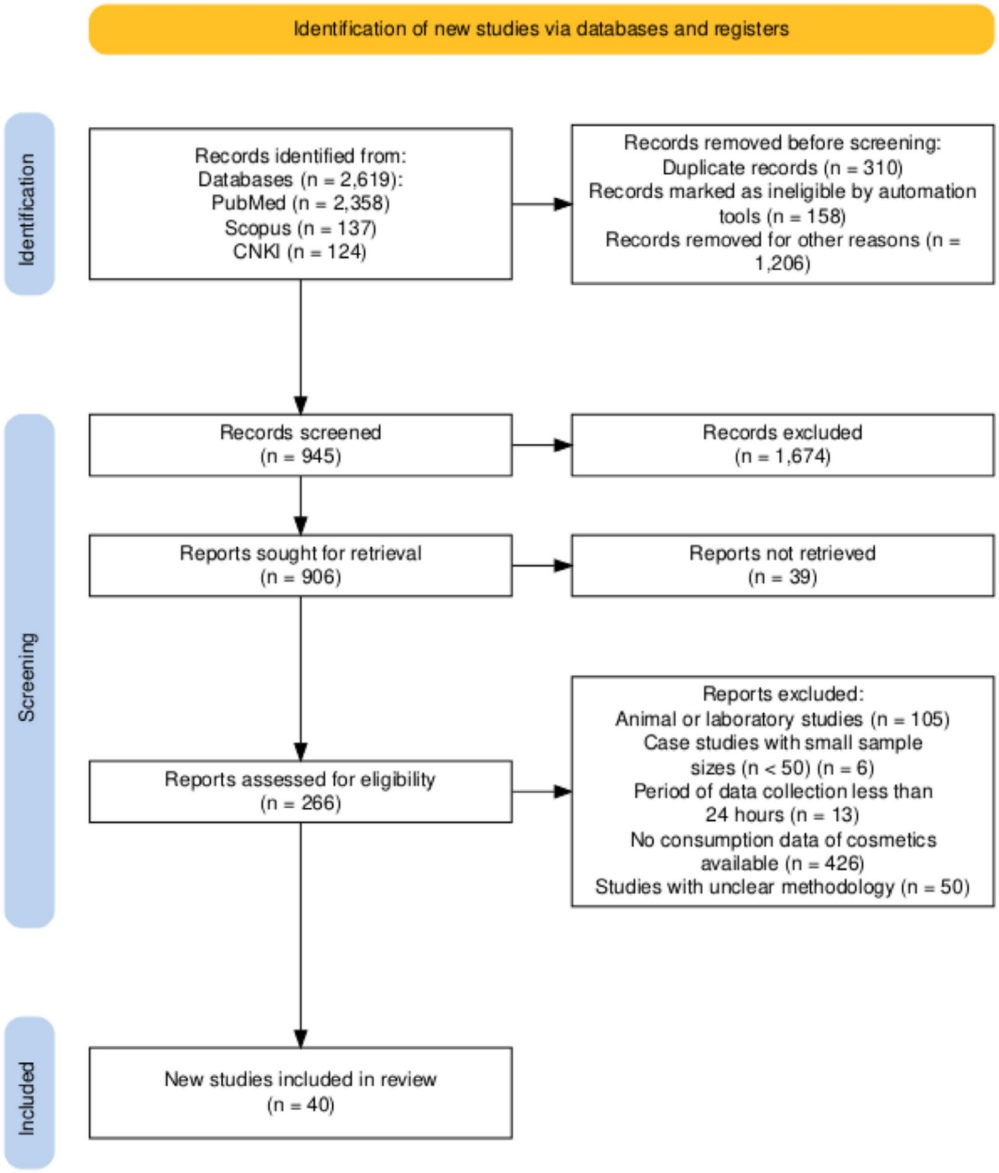
The PRISMA flow diagram of the study.

This systematic review followed the Preferred Reporting Items for Systematic Reviews and Meta‐analyses (PRISMA) guidelines for reporting systematic reviews [5]. A corresponding flowchart was generated as shown in Figure 1 [6].

### 2.2 Search Strategy

PubMed, CNKI, and Scopus databases were retrieved using the following query: (cosmetic product OR personal care product OR beauty product) AND (consumption OR usage pattern OR exposure OR use frequency OR habits). Duplicated, non‐English, and non‐Chinese papers were removed. As cosmetic consumption habits evolve and regulatory policies for cosmetics are constantly updated, only studies published after 2005 were selected for analysis.

### 2.3 Selection of studies

First, articles underwent title screening, with only papers related to cosmetics and potentially containing titles related to the fields of cosmetics consumption or exposure retained. Subsequently, abstract screening was conducted, with only papers detailing the consumption and usage of cosmetic products by human populations selected for further review. A preliminary review of the article structure was then conducted. All publications that did not generate novel human consumption data, as well as those that reported and/or utilized previously published consumption data, were excluded.

Upon successful completion of the preliminary abstract screening process, further in‐depth review and comparative analysis were conducted. This systematic review aimed to synthesize existing research on factors influencing cosmetic consumption. The following inclusion and exclusion criteria were applied to ensure methodological rigor and relevance.

### 2.4 The detailed inclusion criteria were as follows

#### 2.4.1 Study Design

- Empirical studies (encompassing quantitative, qualitative, or mixed‐methods approaches).
- Survey‐based, experimental, or observational studies.
- Systematic reviews and meta‐analyses (provided they include primary data on cosmetic consumption).

#### 2.4.2 Population

- Studies focusing on cosmetic consumers (any race, age, and gender).
- Research on specific demographic groups (e.g., infants and young children, pregnant women).

#### 2.4.3 Outcomes

- Studies reporting cosmetic expenditure, purchase frequency, or influencing factors (e.g., climate, culture, and social media).
- Research measuring psychological or economic drivers (e.g., self‐esteem, disposable income).

#### 2.4.4 Publication Type and Language

- Peer‐reviewed journal articles, theses, and conference papers.
- Publications exclusively in English or Chinese.
- Published between 2005 and 2025 (to ensure relevance to current trends).

### 2.5 The detailed exclusion criteria were as follows

#### 2.5.1 Study Design

- Studies identified as incomplete research papers.
- Non‐empirical studies (e.g., editorials, opinion pieces, theoretical papers).
- Case studies with small sample sizes (*n* < 50) unless qualitative.
- Animal or laboratory studies (irrelevant to consumer behavior).

#### 2.5.2 Population and Scope

- Studies on medical or therapeutic cosmetics (e.g., prescription skincare) unless they include consumer behavior data.

#### 2.5.3 Data Quality and Availability

- Studies presenting unclear methodology (e.g., missing sample size, statistical methods).
- Studies with a data collection period less than 24 h (Short‐term sampling exhibits weaker representativeness).
- Unpublished preprints (unless peer‐reviewed later).
- Duplicate publications (only the most comprehensive version included).

### 2.6 Search results

All identified summaries from the database search were thoroughly reviewed. A total of 2,358 references were retrieved from PubMed, 137 from Scopus, and 22 from CNKI.

Following the application of the inclusion criteria, a total of 38 articles published during January 2005 to December 2025 were included, and the collected references included the following information: the type of study (consumption, epidemiological, modeling, etc.), the period of data collection, the characteristics of the interest population (country, age, sex, number, etc.), the types of cosmetic products studied, the approach(es) of data collection, the usage patterns (percentage of users, frequency of use, amount of products used, daily amount of products used, number of co‐used products, etc.), and/or human exposure parameters to cosmetics [7].

### 2.7 Restricted or Proprietary Data

A portion of the exposure metrics was derived from internal industry reports or commercial databases that are not publicly available due to licensing agreements or confidentiality clauses. Where available, secondary literature or regulatory summaries that reference these datasets have been cited; however, direct source attribution was not feasible in all cases. Therefore, subsequent statistical analysis may be limited.

## 3 Results

Current Status of Cosmetics Usage Pattern Investigation in Different Countries and Regions.

### 3.1 Europe

The European Cosmetics, Toiletry and Perfume Association (COLIPA, now known as Cosmetics Europe) conducted three population exposure surveys to gather cosmetic exposure data from consumers within the European Union.

The first study was conducted between 2004 and 2005, with COLIPA conducting an investigation on the exposure of seven kinds of cosmetics (body lotion, facial moisturizer, toothpaste, shampoo, lipstick, deodorant/antiperspirant spray, and deodorant/antiperspirant non‐spray). The survey involved a total of 44100 households and 18057 individual consumers across five European countries (Denmark, France, Great Britain (GB), the United Kingdom, and Spain). The amount and frequency of cosmetics usage were calculated after 1 or 2 weeks. Data related to cosmetic usage were collected through three complementary databases, and statistical analysis was performed using the Monte Carlo probabilistic method [8].

The three databases included: (1) TNS European Toiletries and Cosmetics Database (ETCD), which surveyed 17,561 adults residing in Denmark, France, Germany, the United Kingdom, and Spain via paper questionnaires, mainly including qualitative information such as daily frequency of use of cosmetics, brands of cosmetics used, and application areas across 12 categories, (2) The Ian Smith Consultancy (ISC) database, commissioned by the European Cosmetics Association to construct ISC in Scotland, included 496 adults who recorded the cosmetics they used within 2 weeks and had the cosmetics weighed at the beginning and after the survey to determine the amount of cosmetics used; (3) The TNS Europanel database, which annually provides purchase information for 44,100 households across 40 European countries where consumers buy cosmetic [9, 10].

The second survey was carried out in 2011. COLIPA used the same method as the previous survey to assess a total of 80,000 families and 14,413 individual consumers across five countries. The survey focused on the exposure to five cosmetic product categories: hair styling products, hand cream, liquid foundation, mouthwash, and shower gel [10]. For the third study in 2012, COLIPA conducted a separate study on deodorant/antiperspirant in spray form [11].

These data are widely used and are also referred to in the “SCCS Notes of Guidance for the Testing of Cosmetic Ingredients and Their Safety Evaluation”, released by the SCCS [4]. However, access to information in the aforementioned databases is limited.

### 3.2 Netherlands

In addition to region‐specific surveys, some countries in the European Union, such as the Netherlands, have also conducted large‐scale surveys.

Between 2011 and 2012, the National Institute for Public Health and the Environment (RIVM) and the Radboud Radelberg Medical Center conducted a cosmetic consumption survey on 516 adults, using a combination of electronic and paper questionnaires to collect qualitative data on the frequency and percentage of use of 32 types of cosmetics. This questionnaire contained general questions regarding demographics, lifestyle, and skin type. The detailed usage patterns of 32 types of cosmetics were assessed using questions regarding the frequency of use and the amount of product used per application. At the same time, photographs were used to visualize the amount of product used in the following product categories: general hygiene, shaving products, hair care, skin care, and tanning products. The photographs displayed three images illustrating an increasing amount of product. This information was used to transform the categorical data provided by the respondents into numerical values for actual exposure calculation. However, for certain cosmetics: deodorant spray, perfume or eau de toilette, aftershave spray, hair spray, eye shadow, mascara, eye pencil, eyebrow pencil, lip pencil, lipstick, lip balm, and nail polish, a visual display of amounts was not meaningful. For these products, alternative questions were developed to describe the amounts used, such as: How often did you spray? Where exactly did you apply the product? How many layers did you apply? The mean amounts used were calculated by weighing the product before and after application. In addition, the questionnaire contained questions regarding the type and brand of the product, the area of the body where the product was applied, the time of day the product was used, the location of use, and the presence of ventilation. All questions concerned product use within the past 6 months, except for tanning products, for which data covered the past year to minimize seasonal influences, providing basic data for subsequent assessment of target chemical exposure in cosmetics [12].

### 3.3 France

Up to now, France has also conducted three large‐scale surveys on cosmetics consumption. Between 2013 and 2015, the French National Agency for Drugs and Health Product Safety (ANSM) initiated a project to evaluate the consumption of cosmetics among French consumers. The study population included infants, children, adults, and pregnant women. A total of nearly 150 kinds of product consumption data were collected, including personal care products, perfume, cosmetics, and baby products.

This project comprised a total of four studies: (1) The first study involved an online questionnaire survey, recruiting 7313 individuals (5657 French adults aged 15–70 years, 1079 children aged 4–14 years, and 395 babies aged 0–3 years). The survey aimed to obtain qualitative data, such as the percentage and frequency of cosmetic use. This enquiry collected current information on the usage patterns of 139 cosmetic products, categorized as general hygiene, skincare, hair styling, hair care, make‐up, fragrances, solar, shaving, and depilatory products. Specific bottom care products were studied for babies under 3 years [13]; (2) The second study aimed to assess the amount per use of cosmetic products consumed at home by the adult, child, and baby French population. A total of 1078 men and women participated in the study, which was performed in four cities in France. This enquiry was performed on 106 cosmetics, including general hygiene, skincare, hair care, hair styling, make‐up, fragrances, solar, shaving, and depilatory, and baby products [14]; (3) The third component of the project, focusing on cosmetic product consumption by the French population, sought to generate Product Exposure Amount data, that is, the amounts of cosmetics applied to the skin among the French population using the raw data collected during the previous enquiry. Exposure data were generated for 69 different cosmetics, classified as products for the hair, face, buccal hygiene, hands, feet, body, shaving, and depilation, sunscreens, as well as products specifically intended for babies. Exposure was calculated using a probabilistic Monte Carlo method [15].

The project involved a large‐scale study involving over 20,000 French participants, and a Monte Carlo probability approach was used to simulate the consumption of cosmetics across France [13, 14, 15]. This project first collected large‐scale qualitative data on the consumption frequency of nearly 150 types of cosmetics for infants, children, adults, and pregnant women in France. This information, initially collected through online questionnaires from 1200 respondents, was subsequently verified and supplemented via telephone surveys. Then, by obtaining information on the usage of each type of cosmetics by the population, the overall cosmetics consumption by the French population was calculated. This study is characterized by its provision of stable and reliable information.

Consumption and exposure data are available for family cosmetic products were notably scarce. The Laboratoire d'Evaluation du Risque Chimique pour le Consommateur (LERCCo) undertook a study to assess the consumption of and exposure to such products among infants, children, and adults. Ten categories of products were studied: shampoo, shower gel, solid soap, cleansing lotion, emollient foam, emollient bath cream, cream, milk, balm, and lip balm. Consumption data were obtained from 2994 participants (789 babies aged 0–3 years, 837 children aged 4–12 years, and 1368 adults aged more than 18 years) included in 87 clinical safety studies. Exposure was assessed using a probabilistic method. The age‐stratified consumption and exposure assessment enhanced the study's robustness, revealing distinct differences, particularly in exposure levels. Infants consistently showed the highest exposure to family products, followed by children and adults. These novel data will be beneficial for safety assessors and agencies in safeguarding consumers [16].

### 3.4 Switzerland

In 2015, ETH Zurich conducted a questionnaire survey in Switzerland on the usage patterns of 12 household care products, five laundry products, and 22 personal care products among 759 cosmetics consumers aged 0–91 in Switzerland. Thirteen categories of personal care products were evaluated for consumer usage using images, and the impact of the Swiss cultural environment, climate, and geographical factors on cleaning and grooming behavior was analyzed [17].

### 3.5 United States

As the world's largest consumer of cosmetics, the United States has a long history of investigating cosmetics consumption and has conducted four surveys to date.

From 2000 to 2005, the Cosmetic, Toiletry, and Fragrance Association (now known as the Personal Care Products Council (PCPC)) spearheaded research at distinct geographical locations within the US, for example, Atlanta, Boston, Chicago, Denver, Houston, Minneapolis, St. Louis, San Bernardino, Tampa, and Seattle. A survey was administered to 360 female consumers aged 19–65 regarding their consumption of 12 types of cosmetics [18]. The survey methodology involved the on‐site distribution of test products to participants, allowing them to use them for 2 weeks [19]. Participants filled out a paper questionnaire to record daily usage information, and the test products were weighed before and after use. Finally, data regarding the percentage of users, frequency of use, and duration of use were obtained. Consumption data for these cosmetics, encompassing factors such as application location and method, were analyzed using a Chi‐square test to ascertain differences across various age groups and geographical regions [20].

In addition, researchers from the University of California conducted a study in 2010 on 30 types of personal care products, including skincare, makeup, hair products, and hygiene products, in 604 households (including children, adults, and the elderly) in California. The survey was conducted in the form of telephone interviews, which included frequency of use, frequency of purchase, product awareness, and preferences. Statistical analysis was conducted on gender, age, education, race, and other aspects in groups [21]. To reduce participant withdrawal rates and recall bias, this group of researchers subsequently used barcode scanning instead of telephone follow‐up to investigate the exposure levels associated with 17 personal care products in 47 households [22].

### 3.6 Korea

Korea is the first Asian country to have conducted large‐scale consumption surveys of its population, with four investigations having been undertaken in the past decade.

In 2012, Seoul National University in Korea conducted a survey on the consumption of five personal care products and five home care products by 816 males and 2517 females from 2500 households in 15 provinces, cities, and districts of Korea. The survey adopted face‐to‐face questionnaire interviews. The participants reviewed their product usage within the first 3 months and estimated the amount used per application based on a graphical comparison approach. Subsequently, 85 households in the Seoul region were further selected for follow‐up, and specific investigations were conducted on the usage environment and the quantity used per application. Information such as brand details, usage frequency, usage location, usage environment, and estimated amount used each time was obtained for consumer goods [23].

In 2014, Tangoku University in Korea conducted surveys across five regions (Seoul, Incheon, Daeju, and Busan). Data on the usage frequency of 31 types of cosmetics were collected from 1800 people aged 15–59 via online questionnaires [24]. In 2015, the same research team conducted a survey on the usage of eight types of makeup among 358 female adults and 79 male adults in the same regions in Korea. The study involved obtaining the frequency and location of makeup use over a 2‐week period through daily records, and a balance was utilized to measure the amount of product consumed before and after the 2‐week duration [25].

In the summer and winter of 2016, the research team investigated 169 female infants and 167 male infants aged 0–36. By examining parents' habits of using cosmetics on infants, the frequency of using seven types of infant cosmetics was recorded daily for 2 weeks, and the amount of use was measured before and after the use of cosmetics [26].

### 3.7 China

The studies conducted in China mainly focused on assessing the exposure to specific substances of safety concern in cosmetic products. All studies were conducted with a relatively small survey population, mainly targeting very restricted cities such as Shanghai and Guangzhou. According to published data, Shanghai has been the site of five such surveys, detailed as follows:

In 2014, the Shanghai Entry Exit Inspection and Quarantine Bureau, in collaboration with Fudan University, conducted a survey focusing on the average monthly consumption of cosmetics among 153 female college students at a university in Shanghai. The survey targeted female college students, and the product categories only included: perfume, hair care products, skin care products, and nail care products. The primary objective was to assess the cumulative exposure risk of exposure to phthalates (widely used plasticizers) via cosmetic use [27]. In the same year, Fudan University collected cosmetics usage data from 242 women aged 17–55 in Shanghai through a questionnaire survey to understand the exposure of the general female population to phthalate compounds through cosmetics, though only partial data on cosmetics usage frequency and amount data were obtained [28]. In 2015, Fudan University conducted a survey on the cosmetic usage frequency of 103 adult women aged 20–55 in Shanghai to evaluate daily skin exposure to phthalates in both women and infants [29]. In 2020, to monitor the risk of manganese in lip and mouth cosmetics, the Shanghai Institute for Food and Drug Inspection conducted a survey on the consumption of lip and mouth cosmetics using questionnaires and weighing usage. Data on the frequency and usage of lip and mouth cosmetics by women aged 20–60 in 28 provinces/municipalities were obtained [30].

To investigate the usage patterns and habits of leave‐on and rinse‐off cosmetic products among female consumers in Shanghai, the Shanghai Center for Disease Control and Prevention carried out the following two studies: First, from August to December 2021, a questionnaire survey was administered to 309 female consumers of leave‐on cosmetics in Shanghai, recording the use of six categories of products, including skin care lotion, essence, skin care cream, face cream, eye cream, and sunscreen, during the 14 day survey period, and using the difference method for weight measurement [31]. Second, between February and October 2023, a questionnaire survey and weighing method were used to study the daily usage and habits of 328 female consumers of rinse‐off cosmetics in Shanghai during a 14‐day survey period, including five rinse‐off categories of cosmetics: shampoo, conditioner, shower, cleanser, and toothpaste [32]. The results showed that the exposure parameters of female consumers of both leave‐on and rinse‐off cosmetics in Shanghai were lower than those in European and American countries and more similar to those in Asian countries such as Japan and Korea. In 2022, to investigate the consumption of facial mask products used by Chinese people, the Shanghai Center for Disease Control and Prevention recruited 175 healthy subjects over 18 years old to investigate the consumption data of facial mask products, such as the frequency of use, the time of each use, and the skin contact area [33].

To obtain exposure parameters and usage patterns for children's cosmetics in China, a survey on cosmetics consumption was conducted in Wuhan from May 2023 to January 2024, targeting 648 children aged 0–12 years old through questionnaire surveys and cosmetics usage diaries [34]. The results included the proportion, frequency, and dosage of 13 types of cosmetics used, exploring the correlation between cosmetics use and the most common combinations of cosmetics used, and comparing them with relevant exposure parameters abroad. Among them, moisturizing cream, shampoo, and buttock cream (for infants and young children) have the highest usage rate; sunscreen has obvious seasonality. Children aged 0–3 years mainly focused on basic care, and school children aged 6–12 years begin to use cosmetic products (such as lipstick and nail polish). Compared to adults, the single application dose for children was generally lower, but the daily usage frequency was higher (such as 1–2 times a day for moisturizers). Compared with the data obtained from two studies in Switzerland, the usage rate of children's cosmetics in Wuhan was relatively low among the pediatric population. Compared with two studies conducted in France, the frequency and amount of cosmetics used were also relatively low.

In 2023, Southern Medical University conducted a cosmetics consumption survey on 2013 Guangzhou consumers. By constructing the “Cosmetics Consumption Survey Questionnaire for All Categories” and evaluating its reliability and validity using a statistical analysis approach, a cosmetics consumption survey was conducted using the questionnaire to analyze the general situation and gender differences of cosmetics consumption among the population in Guangzhou. Furthermore, the standardized data were compared and analyzed with cosmetics consumption survey data in other countries, exploring the differences and relationships between the consumption of cosmetics in Guangzhou and populations abroad [35].

In 2025, the Guangdong University of Technology and Guangdong Institute of Drug Control conducted a survey on 268 males and 412 females to accurately understand the usage patterns of cosmetics among Chinese consumers. This survey adopted the weighing method to accurately determine consumers' actual usage of cosmetics, complemented by photographic documentation to ensure the accuracy and completeness of the information. Exposure factors were obtained for a total of 20 cosmetic products. More than 60% of participants reported using shower gel and shampoo. Approximately 50% of people use facial cleansers, shower gels, shampoos, and conditioners at least once a day. These data hold significant value for conducting a nationwide survey on the exposure level of cosmetics [36].

### 3.8 Japan

Cosmetics consumption surveys in Japan incorporate seasonal factors into their analysis.

In 2015, the Japan Cosmetics Industry Association (JCIA) conducted a survey on the consumption of cosmetics by approximately 300 women in Tokyo. The frequency and usage of 32 types of cosmetics in summer and winter were collected through daily records of respondents over a 2‐week period, complemented by gravimetric measurements (weighing before and after product use). Among them, 268 women participated in the two seasonal surveys [37], indicating seasonal fluctuations in Japan in the amounts of cosmetics applied. Nonetheless, as cosmetics are used throughout the year, it was considered appropriate to combine summer and winter data for safety assessment purposes.

### 3.9 Singapore

Some countries in Asia, such as Singapore, have also begun investigating the consumption of cosmetics.

In 2021, the Singapore Food and Biotechnology Innovation Research Institute (SIFBI) collected data on 16 types of cosmetics from 1296 adults aged 21–64 through online questionnaires and evaluated the product usage per use through photographic documentation [38].

In 2025, the same institution conducted surveys on demographic data and consumption among 494 males and females aged 21–64, investigating 17 products and their frequency of use, thereby contributing to the reduction of data gaps in product exposure within Southeast Asia and facilitating the assessment of differences between Western and Asian populations [39].

### 3.10 Data Types and Survey Approaches

Analysis of the published research data revealed two main types of cosmetic consumption data, namely qualitative data and quantitative data. Qualitative data include user personal information, types of products, skin condition, cosmetics usage, usage location, usage season, etc. These data are typically collected through questionnaires, including paper questionnaires, web‐based questionnaires, and telephone questionnaires. Quantitative data include usage frequency and the amount of cosmetics used. Notably, usage frequency, as semi‐quantitative data, is usually obtained through questionnaire surveys. The precise amount of cosmetics used is usually obtained by one of four methods: pre‐ and post‐use weighing, laboratory weighing, photographic assessment, or indicator prompting.

### 3.11 Qualitative data

#### 3.11.1 Postal questionnaire

The survey questionnaires and related materials were dispatched to the respondents, who were then expected to complete the questionnaires according to the stipulated requirements and return them. This postal questionnaire approach typically resulted in a protracted information feedback cycle, compromised timeliness of data collection, and a diminished questionnaire response rate. With the widespread adoption of the internet, few studies have used this approach in recent years.

#### 3.11.2 Face‐to‐face questionnaire or household survey

The selection of appropriate respondents was carried out according to a predetermined approach. Face‐to‐face interviews were conducted directly, following the questionnaire or survey outline, and the responses of the respondents were meticulously recorded. Alternatively, the questionnaire was distributed to the respondents, with the completion requirements clearly explained, and responses were either collected immediately or retrieved at a later time. During the survey process, relatively long‐term and focused responses were typically maintained by the respondents, contributing to a high valid response rate. No restrictions were imposed on the questions within the questionnaire, and researchers retained control over question skipping or the use of open‐ended inquiries. However, this method placed substantial demands on labor, time, and financial resources. Furthermore, stringent requirements were imposed upon investigators, rendering their management and evaluation somewhat challenging.

#### 3.11.3 Web‐based questionnaire

Web‐based questionnaires were utilized, employing computers and the internet as communication mediums, to ascertain the cosmetics consumption of the population through online questionnaire formats. This approach offers several advantages, including low cost, high production speed, rapid data collection, and straightforward operation, and is currently widely used in consumer survey research.

#### 3.11.4 Telephone survey

A telephone survey approach involves collecting survey data through recorded responses from participants to a questionnaire. This process is conducted by dialing phone numbers and adhering to predefined questionnaire requirements and training protocols. This approach features fast data collection, low cost, a simple investigation process, and relatively low professional requirements for investigators. However, the questions cannot be overly complex, and the success rate of visits is low.

### 3.12 Approaches for quantitative data

#### 3.12.1 Direct weighing approach (Pre–post use weighing)

The weighing approach typically involved respondents completing a paper questionnaire and recording daily usage information, as well as using a scale to measure the amount of various cosmetics used before and after each use. Although this approach is cumbersome to operate, it has high accuracy and detailed data collection and is currently widely used in quantitative research on cosmetics consumption.

#### 3.12.2 Laboratory weighing approach

The laboratory weighing approach involved recruiting interviewees to enter the laboratory, measuring the amount of each type of cosmetic used, obtaining the amount of product used each time, and then converting the number of cosmetics consumed based on the frequency of use. While this approach may not achieve the same level of precision as direct weighing methods, it offers advantages such as low cost, reduced expenditure of manpower and resources, and the capability to rapidly acquire a substantial volume of data.

#### 3.12.3 Visualized photographs approach

By incorporating photographs or indicators into a visualization questionnaire, cosmetics were graded into categories such as “much less,” “less,” “more,” and “much more.” These categories were supplemented by pre‐taken images of the same product category displaying varying amounts. Simultaneously, the cosmetics were weighed to ascertain the quantity of product used per application. The consumption of cosmetics was subsequently converted based on the reported frequency of use. This approach estimated consumption based on usage patterns and frequency, exhibiting less accuracy than direct weighing methods. Furthermore, it presented the challenge of potential deviations from real‐life consumption due to its reliance on usage frequency estimations. Nevertheless, this methodology offered advantages, including low cost and the ability to obtain substantial data rapidly, making it suitable for large‐scale investigations Table 1.

**TABLE 1:** Comparison of approaches for investigating the usage of cosmetics.

Data type	Application	Survey approach	Advantage	Disadvantage
Qualitative data	User personal information, types of products, skin condition, cosmetics usage, usage location, usage season, and usage frequency	Postal questionnaire	Wide applicability, high recovery rate, and sufficient time for respondents to fill out questionnaires	High time cost, difficulty in data processing, difficulty in ensuring confidentiality, and unfavorable for large‐scale investigations
		Face‐to‐face interview	More precise investigation of the population and ready communication	High time consumption, high labor costs, high economic costs, and slow data collection speed
		Telephone survey	Fast effectiveness, low cost, wide coverage, and easy control of survey quality	The investigation time should not be long, the success rate of the interview is low, and it is not possible to access units and individuals without a phone number
		Web‐based questionnaire	Low cost, fast production speed, and fast collection speed	Low data validity and high technical requirements
Quantitative data	The amount of cosmetics used	Direct weighing approach	High accuracy	Complicated operation, low compliance of respondents, and data omissions
		Laboratory weighing approach	Low cost	Estimation of usage based on usage frequency, with deviation from actual usage
		Visualized photographs approach	Low cost, large amount of data obtained in a short time	The accuracy of estimating usage based on the pattern and usage frequency is not as high as that of the weighing approach

### 3.13 Summary of Cosmetics Exposure Parameters in Different Countries

As shown in Table 2, research results on cosmetics exposure in specific countries in Europe, America, and Asia revealed variations in usage patterns for the same types of cosmetics.

**TABLE 2:** Comparison of exposure parameters among regions and countries.

Product	European Union										North America			Asia											
	Europe [8, 9, 10]			France [13, 14]						Netherlands [12]	United States [18, 19, 20]			Korea [23, 25]				China [36]			Japan [37]				Singapore [39]
	Amount per day (g/day)			Frequency (times/day) Adult women (≥ 15 years old)			Amount per application (g/use) Adult women (≥ 15 years old)			Amount per day (g/day)	Amount per day (g/day)			Mass per use (g/use)	Amount per day (g/day)	Amount per day (mg/day) Adult women		Amount per day (g/day)			Amount per day (g/day)				Amount per day (g/day)
	Mean	P50	P95	Mean	P50	P95	Mean	P50	P95	Mean	Mean	P50	P95	Mean		Mean	P95	Mean	P50	P95	Summer		Winter		Mean
																					Mean	P50	Mean	P50	
Perfume	—	—	—	0.98	1	2	0.26	0.22	0.59	0.1	0.53	0.34	1.77	—	—	—	—	0.14	0.16	0.24	—	—	—	—	0.17
Shampoo	6.03	5.50	12.18	0.44	0.36	1	10.40	8.10	25.3	2.4	12.8	10.75	29.08	3.5	3.4	—	—	3.85	3.3	8.88	—	—	—	—	3.92
Hair Conditioner	—	—	—	0.37	0.36	1	10.00	7.50	27.00	2.1	13.77	10.62	33.19	3.0	2.8	—	—	2.63	2.40	5.18	—	—	—	—	2.70
Hair gel	1.91	1.55	4.99	0.58	0.36	1	2.00	1.50	4.30	1.0	3.57	2.71	9.89	—	—	—	—	—	—	—	—	—	—	—	1.04
Face cream	0.91	0.85	1.80	1.05	1	2	0.94	0.68	2.63	0.4	2.05	1.53	3.99	0.86	2.92	—	—	0.78	0.46	2.63	0.35	0.26	0.47	0.36	0.47
Body lotion	4.54	4.56	8.95	0.7	1	2	10.10	8.10	28.00	3.6	8.69	7.63	16.83	—	—	—	—	2.44	2.14	5.45	—	—	—	—	1.57
Eye cream	—	—	—	0.96	1	2	0.157	0.112	0.48	—	—	—	—	—	—	—	—	0.15	0.1	0.044	—	—	—	—	—
Sunscreen	—	—	—	1.41	2	—	2.5	1.5	8	0.4	—	—	—	—	—	—	—	0.61	0.41	1.83	—	—	—	—	0.2
Cleanser	—	—	—	1.07	1	2	2.94	2.68	6.44	1.1	4.06	3.25	9.93	1.1	1.8	—	—	0.94	0.62	2.55	—	—	—	—	—
Bath lotion	11.34	10.89	22.77	0.83	1	2	10.20	8.00	23.20	4.5	14.5	12.9	29.1	2.9	2.7	—	—	4.91	3.93	10.40	—	—	—	—	7.35
Toothpaste	2.09	2.10	2.96	1.59	2	—	1.28	1.44	3.20	2.2	—	—	—	0.9	2.2	—	—	1.25	1.12	2.44	—	—	—	—	1.9
Foundation	0.23	0.17	0.64	0.74	1	1	0.06	0.04	0.16	0.2	0.67	0.45	2.18	—	—	162.3	376.76	0.17	0.18	0.34	0.16	0.12	0.17	0.15	0.06
Make‐up remover	—	—	—	1.01	1	2	4.39	3.53	10.19	1.5	—	—	—	—	—	—	—	1.95	0.98	5.15	—	—	—	—	—
Hand cream	1.06	0.87	2.74	0.88	1	2	1.12	0.88	2.73	0.4	—	—	—	—	—	—	—	0.56	0.53	1.04	1.53	1.30	1.72	1.41	0.4
Liquid soap	—	—	—	1.18	1	2	2.42	1.99	4.90	—	—	—	—	—	—	—	—	—	—	—	—	—	—	—	—
Lip balm	—	—	—	1.19	1	2	0.016	0.011	0.049	0.009	—	—	—	—	—	22.41	70.5	0.08	0.02	0.35	—	—	—	—	—
Lipstick	0.025	0.017	0.07	0.97	1	2	0.008	0.007	0.016	0.004	0.024	0.013	0.087	—	—	22.41	70.5	—	—	—	—	—	—	—	—
Eye shadow	—	—	—	0.44	0.3	1	0.009	0.006	0.020	—	0.04	0.01	0.96	—	—	7.38	17.17	0.01	0.005	0.03	—	—	—	—	—
Mascara	—	—	—	0.37	0.3	1	0.017	0.158	27.00	0.008	—	—	—	—	—	14.23	39.77	—	0.07	—	—	—	—	—	—
Eyebrow pencil	—	—	—	0.58	0.3	1	0.003	0.001	0.007	0.0003	—	—	—	—	—	2.5	7.76	0.02	0.01	0.07	—	—	—	—	—
Nail polish	—	—	—	1.05	1	2	0.94	0.68	2.63	0.04	—	—	—	—	—	—	—	—	—	—	—	—	—	—	—
Nail polish remover	—	—	—	0.7	1	2	10.10	8.10	28.00	0.03	—	—	—	—	—	—	—	—	—	—	—	—	—	—	—

**FIGURE 2:**
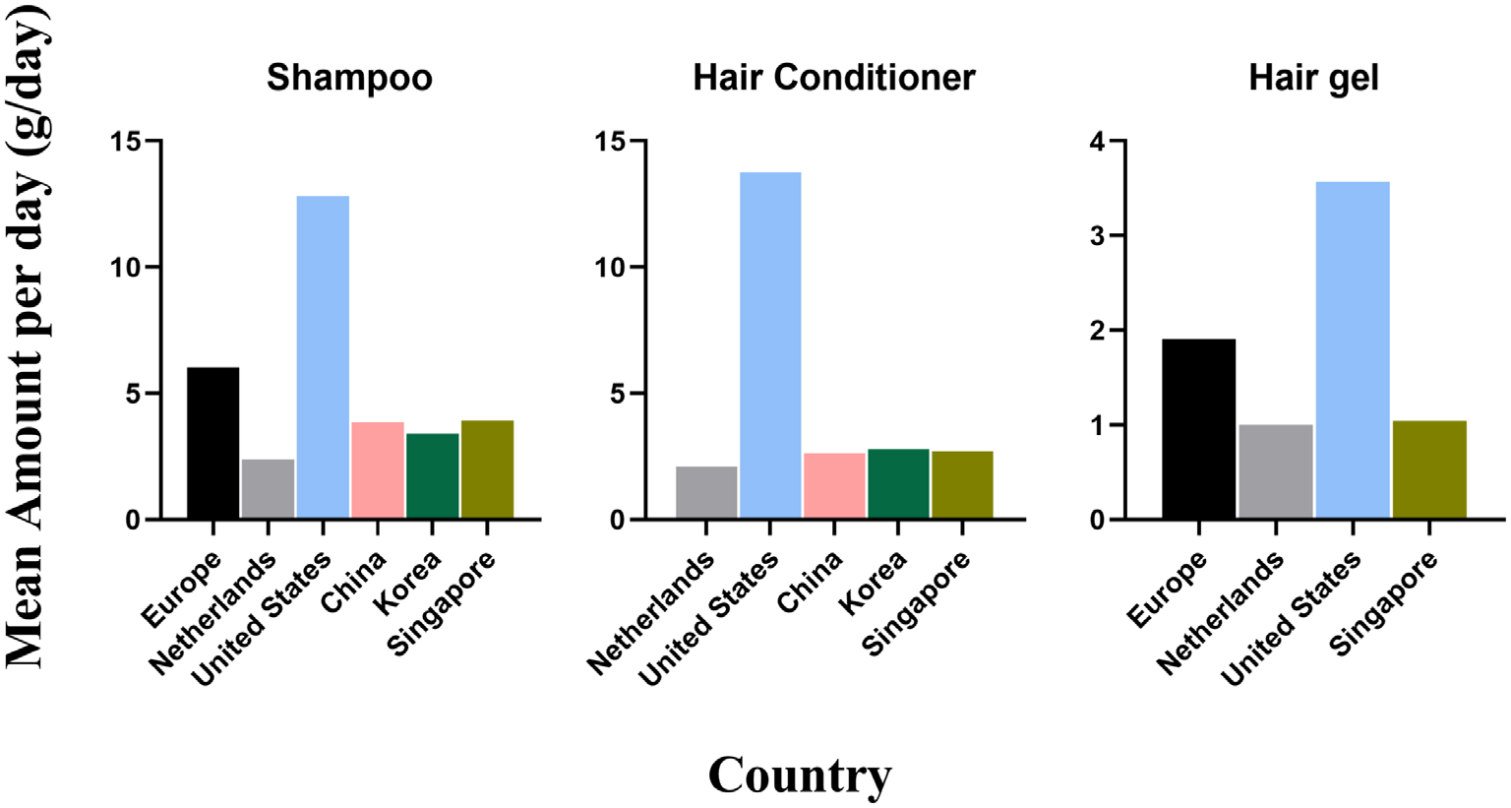
Comparison of the daily mean hair care product usage (amount of use) in Europe, the Netherlands, the United States, China, Singapore, Korea, and Singapore.

Figure 2 illustrates differences in the mean amount per day of hair care products among consumers from Europe, the Netherlands, the United States, China, Korea, and Singapore. The United States consumers exhibited significantly higher daily consumption of shampoo, conditioner, and hair gel products compared to other countries, with values of 12.8, 13.77, and 3.57 g/day, respectively. European consumers have the second highest daily consumption of shampoo and hair gel products, at 6.03 and 1.91 g/day, respectively. Other countries showed similar consumption amounts for these products. As the country with the highest mean amount per day of hair care products, the United States recorded shampoo consumption levels that were 2.12 times that of the EU, 3.32 times that of China, 3.76 times that of Korea, and 3.27 times that of Singapore. Conditioner usage in the United States was 6.56 times that of the Netherlands, 5.24 times that of China, 4.59 times that of Korea, and 5.1 times that of Singapore. Hair gel consumption in the United States was 1.87 times that of the EU, 3.57 times that of the Netherlands, and 3.43 times that of Singapore.

**FIGURE 3:**
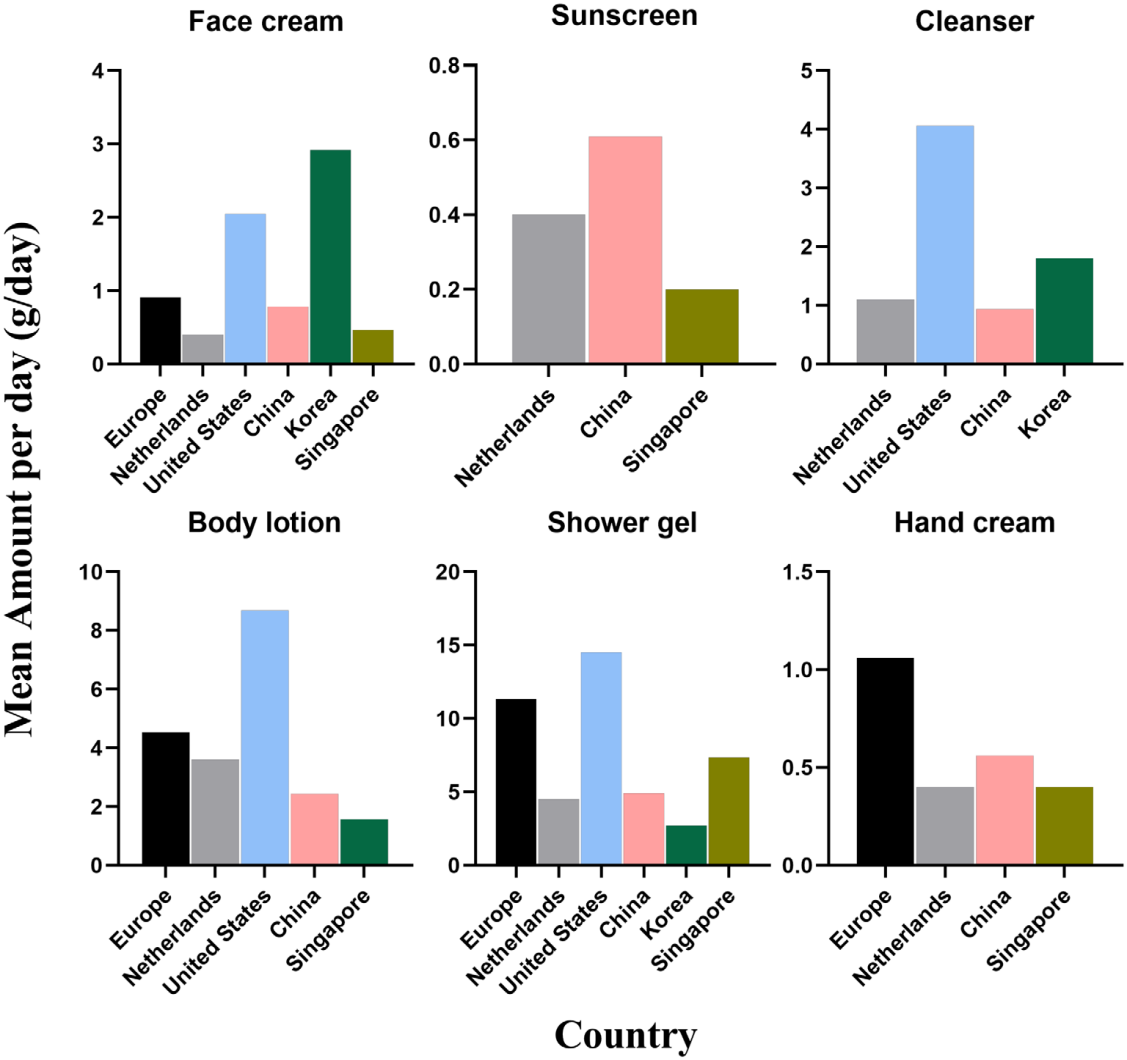
Comparison of the daily mean Skin Care product usage (amount of use) in Europe, the Netherlands, the United States, China, Singapore, Korea, and Singapore.

Figure 3 demonstrates variations in the mean amount per day of skincare products across the six countries. Korean consumers had the highest daily consumption of facial cream at 2.92 g/day, which was 3.21 times that of the EU, 7.3 times that of the Netherlands, 1.42 times that of the United States, 3.74 times that of China, and 6.21 times that of Singapore. Chinese consumers recorded the highest daily sunscreen usage at 0.61 g/day, which was 1.53 times that of the Netherlands and 3.05 times that of Singapore. The United States consumers led in the daily usage of facial cleanser, body wash, and body lotion, with values of 4.06, 15.5, and 8.69 g/day, respectively. European consumers ranked second in the daily usage of facial cream, body wash, body lotion, and hand cream, at 0.91, 11.34, 4.54, and 1.06 g/day, respectively.

**FIGURE 4:**
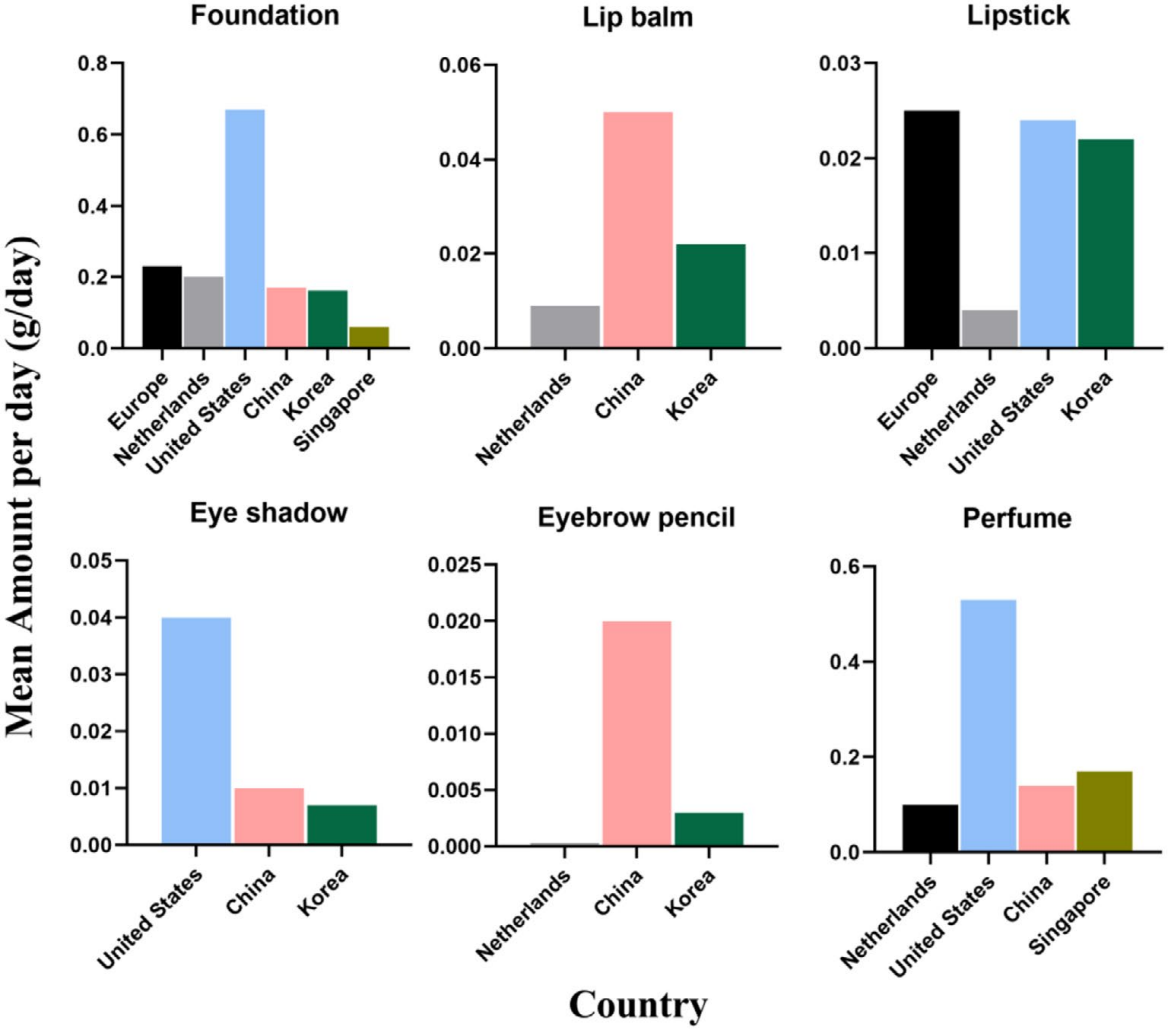
Comparison of the daily mean makeup product usage (amount of use) in Europe, the Netherlands, the United States, China, Singapore, Korea, and Singapore.

Figure 4 highlights differences in the consumption of makeup products across countries. For foundation cosmetics, the United States had the highest consumption at 0.67 g/day, which was 11.17 times that of Singapore. The United States also led in eyeshadow and fragrance product consumption, at 0.04 and 0.63 g/day, respectively. China recorded the highest daily usage of lipstick and eyebrow pencil products, at 0.05 and 0.02 g/day, which were 2.27 times and 6.67 times that of Korea, respectively.

**FIGURE 5:**
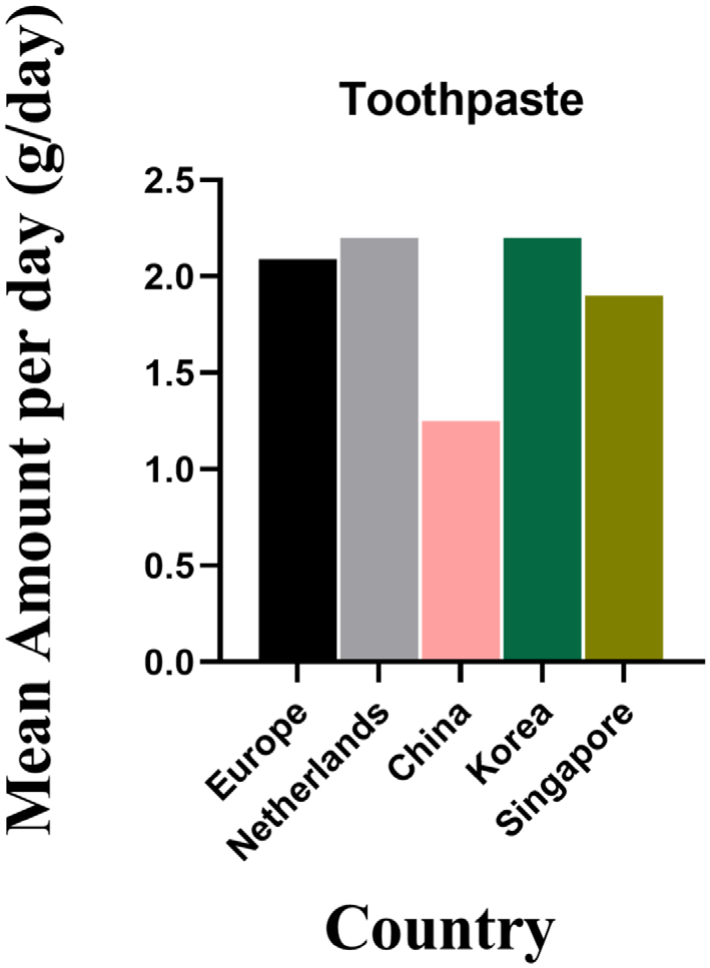
Comparison of the daily mean Oral products usage (amount of use) in Europe, the Netherlands, the United States, China, Singapore, Korea, and Singapore.

Figure 5 presents data on oral care product consumption. China had the lowest toothpaste usage at 1.25 g/day, while other countries showed similar consumption levels: 2.09 g/day in Europe, 2.2 g/day in the Netherlands, 2.2 g/day in Korea, and 1.9 g/day in Singapore.

**FIGURE 6:**
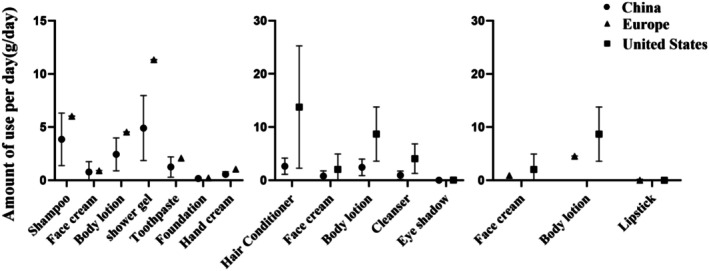
Compared to the median daily use amounts in China, Europe, and the United States.

Figure 6 displays the median and standard deviation of daily cosmetic usage in China, Europe, and the United States. The data reveal differences in median daily consumption between China and Europe. European consumers used 1.57 times more shampoo, 1.17 times more facial cream, 1.86 times more body wash, 2.31 times more toothpaste, 1.67 times more foundation, and 1.89 times more hand cream than their Chinese counterparts. Similarly, differences were observed between China and the United States, with American consumers using 5.24 times more conditioner, 2.63 times more facial cream, 3.56 times more moisturizer, 4.32 times more facial cleanser, and 4 times more eyeshadow than Chinese consumers. A comparison of median daily consumption between Europe and the United States showed that Americans used 2.25 times more facial cream and 1.91 times more body lotion, while lipstick consumption in Europe was 1.04 times that of the United States.

These differences highlight regional disparities in consumer behavior. Similar variations in exposure levels were observed among countries for other types of cosmetics. Overall, the data indicate significant heterogeneity in product usage between and within populations.

## 4 Discussion

This paper summarizes commonly used methods for investigating cosmetic exposure parameters. A comprehensive literature review on existing exposure assessment approaches reveals two primary methods for investigating cosmetic consumption: (1) Survey‐based methods, distributed either in‐person or electronically, can be enhanced with visual aids but are susceptible to low response rates for electronic surveys and potential recall bias due to self‐reporting, (2) Gravimetric analysis involves precise measurement of cosmetic product mass before and after each application, alongside usage frequency, thereby providing detailed records, but it is time‐consuming and costly, requiring high compliance from the subjects. When conducting new research on the use of cosmetics, appropriate research approaches should be selected based on the sampling survey approach, the survey population and sample size, and the survey content.

We previously conducted a survey on the consumption of cosmetics among the population in Guangzhou, which was administered in the form of an electronic questionnaire [35]. The survey collected data on the consumption proportion and weekly frequency of 89 cosmetics, as well as the unit area consumption and weekly consumption of 24 cosmetics. The included cosmetics were classified according to their different parts of action and dosage forms, and the consumption frequency and proportion of cosmetics with different parts of action and dosage forms were discussed. Using gender as a grouping variable, the differences and connections in the frequency and quantity of cosmetics consumption between males and females were discussed. The methodology employed in this survey involved the design and distribution of electronic questionnaires to comprehensively collect information on cosmetic consumption frequency and quantity. The resulting data were then quantified using metrics such as consumption rate (%), frequency (times/week), unit area consumption (mg/cm^2^), and weekly consumption (g). Formal investigations were conducted across various districts of Guangzhou, utilizing chi‐square and Mann–Whitney U tests to compare differences in consumption rate, frequency, and quantity between distinct gender groups. Besides, a comparative analysis was performed between the cosmetic consumption patterns of the Guangzhou population and those reported in other countries. The comprehensive description of these general cosmetic consumption patterns among the Guangzhou population is critical for providing data support for cosmetic safety assessments, thereby significantly contributing to consumer safety and fostering the development of the cosmetics industry [34].

Variations in cosmetic consumption parameters across different countries/regions can be attributed to several factors:

Gender differences in the research population. Women typically prioritize facial makeup, cleaning, and care (including eye and lip products), while men place more emphasis on hair styling and general body hygiene (including hair and face). A 2012 research report from the Netherlands revealed higher percentages of female users for nearly all personal care products examined, except for shaving products [12]. A survey conducted in Switzerland showed that, apart from shaving products and aftershave, the proportion of adult female users among all surveyed personal care products surpassed that of adult male users, with hair dye exhibiting the highest usage among adult females [17]. The Survey Report on the Consumption of Cosmetics among the Guangzhou Population revealed that women's consumption rates for skin care products, such as hand cream and color cosmetics, such as liquid foundation, were significantly higher than those of men, while no significant gender difference was found for personal care products like toothpaste [35]. The proportion, frequency, and unit area consumption of most personal care and makeup products consumed by women are reportedly significantly higher than those of men [13], establishing them as the primary drivers of cosmetic consumption.

Besides, cultural differences and customs exert a profound impact on cosmetic consumption behavior since different countries or regions have varying aesthetic standards. For instance, in many Western countries, healthy bronze skin is considered a symbol of beauty, leading to the popularity of tanning products. At the same time, individualistic culture emphasizes self‐expression, which has given rise to a more diverse range of makeup styles, from natural, nude makeup to dramatic looks. In many East Asian countries, fair skin is considered the standard of beauty, and consumers are more inclined to use whitening products and high SPF sunscreen. For example, the makeup styles in Korea and Japan emphasize clear and natural aesthetics, supported by a well‐established daily makeup etiquette and a wide variety of cosmetic product forms. In South Asia, heavy eye makeup and brightly colored lip makeup are prevalent, and many people prefer to use traditional natural ingredients such as herbal extracts from mulberry trees and rose water. Besides, French consumers use more perfume than Dutch consumers, potentially reflecting a more open attitude toward beauty and hairdressing and better integration of makeup into daily life. Studies on skincare products in Japan indicate that, compared with the studies in the West, larger amounts of lotions and emulsions were applied, whereas lower amounts of cream and essence were applied in Japan. Furthermore, a Wilcoxon signed‐rank test comparing the median daily consumption of eight cosmetic products among American women (as an overall median) with Guangzhou female survey samples revealed that the median daily consumption of cosmetics by the American population was significantly higher than that of the Guangzhou population [35]. Thus, these findings suggest that localized investigations are necessary to accurately reflect the real‐life usage patterns of cosmetic products, which may enhance and complement existing information to facilitate more sophisticated risk assessments.

Climatic conditions are now understood to significantly influence cosmetic consumption patterns. In hot climates, where the skin is prone to sweating and increased oil production, consumers exhibit a preference for oil‐control, waterproof, and durable cosmetics, such as durable foundation makeup and mattifying powders. The low temperature climate induces skin dryness and sensitivity, leading to a predilection for high moisturizing products, such as hydrating liquid foundations, emollient lipsticks, and oil‐rich skin care products. Regarding humidity, a humid environment typically correlates with oily and acne‐prone skin, prompting consumers to opt for lightweight, oil‐controlling, and acne‐preventing cosmetics, such as toners, sheer foundations, and oil‐free skin care products. Dry climates exacerbate skin dehydration and flaking, driving demand for intensely moisturizing products, such as hydrating essences, rich face creams, and moisturizing facial masks. Areas with strong sunshine exhibit a higher demand for sun protection, leading to increased usage of high‐SPF sunscreens, foundations with integrated SPF, and protective sprays; concurrently, antioxidant and reparative skincare products gain popularity to mitigate UV‐induced skin damage. Seasonal changes are widely thought to play a major role. Hot and humid summers favor fresh, oil‐controlling, and sun‐protective cosmetics. In cold and dry winter, consumers need highly moisturizing cosmetics, such as rich face cream, hydrating essences, and hand creams. In spring, given the increase in allergens, consumers may choose anti‐allergic and soothing options. Climate has a significant impact on the use of cosmetics, as different temperatures, humidity, sunlight, and seasonal changes can alter the condition and needs of the skin, thereby affecting consumers' choices and usage habits of cosmetics [37]. Understanding the impact of climate on the use of cosmetics can help cosmetics companies develop and promote products more effectively, meeting the needs of consumers in various climate conditions. Climate conditions exert a discernible impact on cosmetic consumption, with tropical countries exhibiting significantly higher consumption frequency of shampoo, facial cleansers, and other cleaning products compared to temperate countries, indicating that different climate conditions may affect local consumers' cosmetics consumption. This is further supported by Japanese research, which revealed a significant difference in the consumption of cleaning cosmetics between winter and summer.

Regulatory policies and industry standards have a significant impact on the global cosmetics market. The EU's Registration, Evaluation, Authorization and Restriction of Chemicals (REACH) regulation imposes restrictions on cosmetic ingredients, resulting in increased innovation costs for products in the European market; however, it also contributes to the development of leading global safety standards. In Japan, the Pharmaceutical Affairs Law (PAL) governs the manufacturing and distribution of medical devices and pharmaceutical products. The United States enacted the Modernization of Cosmetics Regulation Act (MoCRA) in 2022, updating its regulatory framework for cosmetics. At the same time, the newly issued Regulations on Supervision and Administration of Cosmetics in China in 2020 provide robust mechanisms for industry regulation. These regulatory frameworks and their respective agencies play a key role in ensuring cosmetic safety, establishing ingredient restrictions, dictating labeling requirements, and controlling market access. Indeed, the rigor and emphasis of regulations vary across different countries.

Economic factors significantly influence the capacity of the cosmetics market. Developed economies consistently demonstrate substantially higher per capita cosmetics expenditures compared to developing countries. For example, an epidemiological study conducted on African adults living in Ethiopia [40] revealed a substantially lower percentage of cosmetics users compared to the Netherlands or France [12, 13]. Besides, the number of products used daily was lower than that reported in the French population [13].

Social psychology and media influence exert a considerable influence. Modern communication methods have accelerated the diffusion of consumer attitudes. The widespread penetration of social media and the globalization of beauty blogger culture, prominently exemplified by platforms like YouTube/TikTok, have contributed to an increase in per capita usage of skincare products.

Technological innovation and industrial ecology have been established as critical drivers, since research and development capabilities directly determine the degree of product differentiation and consumer choice.

The investigation of exposure parameters of cosmetic usage is an essential part of cosmetic safety evaluation. Given the unique cultural and economic characteristics of each country, cosmetic consumption or exposure survey data from one nation is not directly transferable or suitable for reference in the safety evaluation of cosmetics in other countries.

When conducting research on cosmetic consumption across diverse regions and countries, it is imperative to select a representative sample of subjects, stratified according to population proportion within each region. The chosen methodology should aim to capture a comprehensive range of parameters relevant to cosmetic exposure, including behavioral, physiological, and environmental factors. This rigorous approach enables a thorough understanding of cosmetic usage patterns that closely approximate real‐life conditions within the scope of the survey, thereby establishing a robust foundation for cosmetic safety evaluations.

## 5 Conclusion

In conclusion, this systematic review highlights the essential role of investigating consumer usage patterns and exposure parameters in a thorough safety assessment of cosmetic products. The current research summarizes the published literature ranging 2005–2025 to reflect various survey methodologies, detailing their merits and limitations in evaluating user behavior, frequency of use, environmental conditions, and regional demographic differences; all of these might give references for future investigations that aim to obtain usage data within an area or country. After conducting investigations using these methods, effective risk management strategies can be implemented, thereby ensuring the safety of cosmetic products and protecting consumer health.

## 6 Impact statement

This review provides a comprehensive overview of the current state of surveys on the exposure parameters of cosmetics consumers in China and abroad, analyzes the regional differences in research on the usage patterns of cosmetics, summarizes the advantages, disadvantages, and applications of the survey methods, and highlights the necessity of investigating cosmetics consumption for fit‐for‐purpose regulatory applications. Furthermore, the review offers recommendations for filling key data gaps.

## Ethics Statement

The authors have nothing to report.

## Conflicts of Interest

The authors declare no conflicts of interest.

## Data Availability

The data that support the findings of this study are available from the corresponding author upon reasonable request.
