# Correction to “Population‐Based Cosmetics Exposure Parameters: Survey Approaches and Cosmetics Usage Results From Different Countries”

**DOI:** 10.1111/jocd.70571

**Published:** 2025-12-02

X. Yin, Z. Li, J. Fu, et al., “Population‐Based Cosmetics Exposure Parameters: Survey Approaches and Cosmetics Usage Results From Different Countries,” *Journal of Cosmetic Dermatology* 24, no. 10 (2025): e70471, 10.1111/jocd.70471.

The funding information “This work was supported by the China Association of fragrance flavor and Gosmetie Industries (CAFFCA), National Key Scientific Instrument and Equipment Development Projects of China during the 14th Five‐year Plan Period (2022YFF0711100), Industry Research Project on Population‐based Cosmetics Exposure Investigation (Phase l) of China Association of Fragrance Flavour and Cosmetic Industries.” was incorrect. This should be: “The work was supported by the Industry Research Project on Population – based Cosmetics Exposure Investigation (Phase I) of China Association of Fragrance Flavour and Cosmetic Industries and the National Key Scientific Instrument and Equipment Development Projects of China during the 14th Five‐year Plan Period (Grant No.2022YFF0711100).”

We apologize for this error.

